# Advancing Academic Cancer Clinical Trials Recruitment in Canada

**DOI:** 10.3390/curroncol28040248

**Published:** 2021-07-28

**Authors:** Rebecca Y. Xu, Diana Kato, Gregory R. Pond, Stephen Sundquist, James Schoales, Saher Lalani, Janet E. Dancey

**Affiliations:** 1 Canadian Cancer Clinical Trials Network, Toronto, ON M5G 0A3, Canada; Rebecca.Xu@oicr.on.ca (R.Y.X.); Diana.Kato@oicr.on.ca (D.K.); Stephen.Sundquist@oicr.on.ca (S.S.); James.Schoales@oicr.on.ca (J.S.); Saher.Lalani@oicr.on.ca (S.L.); 2 Department of Oncology, McMaster University, Hamilton, ON L8S 4L8, Canada; gpond@mcmaster.ca; 3 Canadian Cancer Trials Group, Queen’s University, Kingston, ON K7L 3N6, Canada

**Keywords:** Canadian cancer clinical trials, patient recruitment, funding, academic cancer trials

## Abstract

The Canadian Cancer Clinical Trials Network (3CTN) was established in 2014 to address the decline in academic cancer clinical trials (ACCT) activity. Funding was provided to cancer centres to conduct a Portfolio of ACCTs. Larger centres received core funding and were paired with smaller centres to enable support and sharing of resources. All centres were eligible for incentive-based funding for recruitment above pre-3CTN baseline. Established performance measures were collected and tracked. The overall recruitment target was 50% above pre-3CTN baseline by Year 4. An analysis was completed to identify predictive success factors and descriptive statistics were used to summarize site characteristics and outcomes. From 2014–2018, a total of 11,275 patients were recruited to 559 Portfolio trials, an overall increase of 59.6% above pre-3CTN baseline was observed in Year 4. Twenty-five (51%) adult centres met the Year 4 recruitment target and the overall recruitment target was met within three years. Three factors that correlated with sites’ achieving recruitment targets were: time period, region and number of baseline trials. 3CTN was successful in meeting its objectives and will continue to support ACCTs and member cancer centres, monitor performance over time and seek continued funding to ensure success, better trial access and outcomes for patients.

## 1. Background

Clinical trials are essential for advancing scientific knowledge and identifying better treatment for patients. Canadians have long benefited from access to and results from practice-changing cancer clinical trials sponsored by the academic investigator community [1,2,3]. However, that access is threatened by increased complexity of ethical and regulatory processes, rising costs, and limited supports available for the conduct of academic trials [4]. Limited capacity to participate in academic sponsored trials has resulted in greater prevalence of industry sponsored clinical trials that are designed to lead to the approval of new drugs [5]. Trials to address other important practice-changing questions are delayed or unable to be addressed.

**Figure 1:**
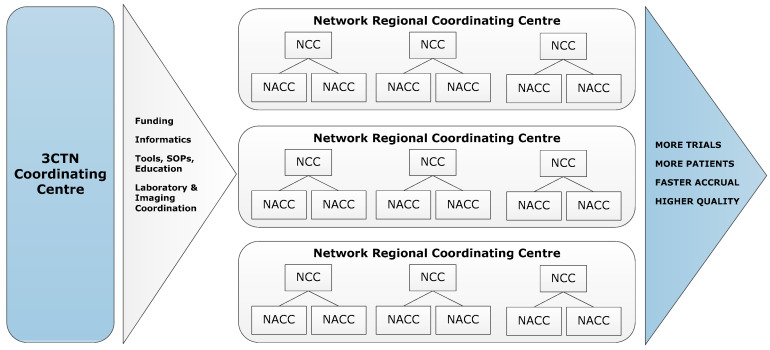
3CTN organizational framework.

To address the impediments to academic clinical trial activities, the Canadian Cancer Research Alliance (CCRA) 2011 report on the status of clinical trials in Canada recommended the establishment of a pan-Canadian network that would provide funding and infrastructure to support the conduct of academic clinical trials [1]. In 2014, the Canadian Cancer Clinical Trials Network (3CTN; ‘the Network’) was established in collaboration with the Ontario Institute for Cancer Research (OICR), Canadian Cancer Trials Group (CCTG) and Network of Networks (N2). A total funding envelope of CAN 22 M raised was provided from 13 national and provincial charities, and public agencies. The Network has four components: (a) Coordinating Centre in Toronto serving as the administrative hub; (b) Network Regional Coordinating Centres (NRCC) that cover Canadian provinces and regions; (c) larger Network Cancer Centres (NCC); (d) smaller Network Affiliated Cancer Centres (NACC) that are linked to NCCs (Figure 1). The Network is comprised of 66 member institutions committed to the following goals and objectives for its initial four-year strategic plan:

- To improve patient access to academic clinical trials;
- To improve site performance of academic trials;
- To improve the trial environment for the conduct of academic clinical trials through collaboration and facilitation of important national trial initiatives;
- To demonstrate impact of the Network and academic trials on the Canadian Health System.

The majority of adjusted funds were allocated to core staffing at Cancer Centres’ clinical trial units, to support costs of additional resources necessary for conducting academic trials [6,7]. Funding was provided to NRCCs for regional coordination to realize Network goals and objectives. NCCs received core funding from provincial funders to support academic cancer clinical trial (ACCT) recruitment based on population served. The smaller NACCs were paired with NCCs to form local-regional nodes to enable better support and sharing of resources and NCCs were encouraged to share a portion of their core funds with affiliated centres. In addition, both NCCs and NACCs were each eligible to receive incentive-based funding for recruitment to ACCTs above their defined, pre-3CTN baseline. Further to providing funding for trial recruitment, 3CTN provided a coordinated focus on improving ACCT trial activities, supported trial best practices, the development and implementation of patient involvement strategy for the Network and its centres, as well as the development of communication and knowledge transfer activities to meet its four-year strategic goals [8,9,10]. This paper aims to describe the impact of the Network to ACCT recruitment and identify predictive success factors for adult Network sites after 3CTN’s first four-year term.

## 2. Methods

### 2.1. Development of Portfolio Trials and Pre-3CTN Baseline

3CTN aims to support the ‘right’ trials as well as doing trials right to ensure that its limited resources are directed to support research priorities and ensure that these trials are conducted efficiently [11]. A cornerstone of 3CTN’s strategy has been the creation and continuous development of a portfolio of ACCTs that meet the following eligibility criteria: (a) interventional oncology; (b) academically sponsored; (c) open to multiple Canadian sites; (d) funded independently of 3CTN; (e) peer reviewed by external reviewers. The Portfolio contains a robust mix of trial types, design characteristics and potential impacts across cancer types. Established performance measures, such as patient accrual and trial activation timelines are routinely tracked and regularly reported to show trends in the ACCT environment and cancer centre trial participation to support evaluation of 3CTN impact.

### 2.2. Accrual

To show an increase in recruitment to ACCT, establishing an accurate baseline from Network sites was required. Accrual data for trials that met the defined Portfolio eligibility criteria were obtained from each site for 2011 to 2013. Pre-3CTN baselines for each site were derived from the average annual totals, with the aggregate total for all sites forming the pre-3CTN baseline for Canada. Incremental annual targets were then created to support achievement of a 50% increase in overall recruitment above pre-3CTN baseline for adult Network sites by the end of the four-year period.

### 2.3. Portfolio and Recruitment Data Collection and Analyses

Key data abstracted for each trial entered into the Portfolio were sample size, phase, and trial design features such as randomization, treatment line and categories of interest (e.g., immunotherapy, precision medicine etc.). In addition, 3CTN developed a trial complexity rating system and potential impact categories that were applied to all incoming Portfolio trials [11,12]. To analyse the changes in the ACCT environment, trends and changes in Portfolio trial composition were compared to pre-3CTN baseline (2011–2013).

Portfolio trial and recruitment data were collected on a quarterly basis from Network sites between 1 October 2014–31 March 2018. Progress to annual recruitment targets and percentage growth above pre-3CTN baseline were tracked and sites meeting Year 4 recruitment target (≥50% increase) identified. Further data analysis was conducted to identify contributing success factors such as site type (NCC, NACC), size (large, medium, small) and access to core funding from 3CTN. The initial time point of 1 October 2014 included data from 36 adult sites with an additional 11 sites included on 1 October 2015 and one site on 1 March 2016.

Descriptive statistics were used to summarize site characteristics and outcomes. The percent increase in accrual over baseline was calculated as the (number of patients accrued in a given quarter/baseline accrual (by quarter) − 1) × 100%. Logistic regression analysis was used to examine factors potentially prognostic of attaining the year 4 accrual target. Repeated measured methods (generalized estimating equations—GEEs) were used to examine factors potentially prognostic of percent increase in accrual by quarter over baseline. These analyses accounted for time (i.e., quarter) in the analysis. Given the limited statistical power available, factors included in the multivariable models were selected based on clinical expertise and to avoid the potential of collinearity. Analyses were two-sided and statistical significance was defined at the α = 0.05 level.

## 3. Results

### 3.1. Comparison of Portfolio Trials and Pre-3CTN Baseline Trial Activity 

**Figure 2:**
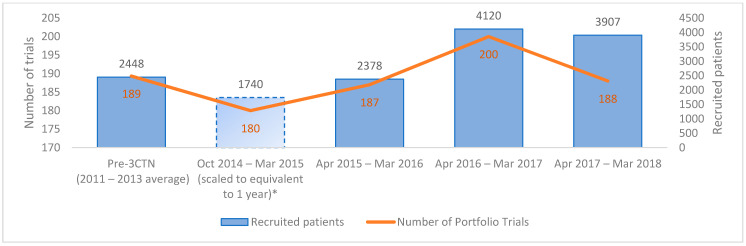
Number of active Portfolio trials and patients recruited at adult member institutions. * Member institutions reported recruitment data as of 1 October 2014.

During the review period, 3CTN supported a mean of 224 Portfolio trials per year. From 2014–2018, a total of 11,275 patients were recruited to 559 Portfolio trials, an overall increase of 59.6% above pre-3CTN baseline was observed in Year 4 (Figure 2). A total of 41 (84%) adult Network sites earned incentive-based funding for accruals above pre-3CTN baseline. Despite the challenging trial environment, 3CTN member institutions contributed to an overall increase in recruitment to ACCTs and met the overall Network recruitment target within three years.

### 3.2. Recruitment Data Analysis of Site Characteristics and Outcomes

Table 1 provides the breakdown of Network site characteristics and outcomes and Table 2 is a comparison of accrual site performance and Portfolio trial composition between pre-3CTN baseline and Year 4. Table 3 provides recruitment performance by quarter. From 1 October 2014–31 March 2016, the mean accrual was between 12–15 patients per site per quarter, however, from 1 April 2016–31 March 2018 the mean accrual was higher, between 17–24. Similarly, a third or less sites met their accrual in the first two years, com-pared with over half of sites in the last two years. 

Results of the GEE analysis for the percentage change in accrual per quarter are presented. No interaction between region and time (*p* = 0.80) was observed. In the multivariable analysis (Table 4), time period, region and number of baseline trials were statistically significant (*p*-value < 0.05) prognostic factors. Larger increases in accrual were observed over time, in Western Canada and Ontario, and among institutions with fewer baseline trials.

**Figure 3:**
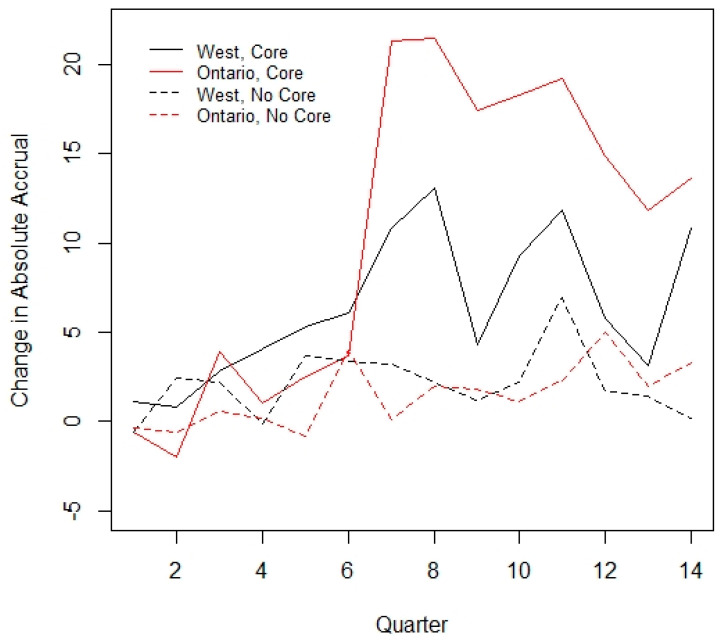
Interaction between core funding and time.

No interaction between core funding and time throughout the period was observed (*p* = 0.38). This indicates that the slope of accrual was similar between those sites that received core funding and those that did not. However, there was a relationship between core funding and region; notably, no site in Quebec received core funding during this time period. A supportive analysis performed excluding the Quebec sites gave similar results (analysis not included below, see Figure 3). Despite not being statistically significant, Western Canada and Ontario sites that received core funding had a marked increase in mean accrual starting in Year 3, which was not observed in the non-core funded sites. A further subgroup analysis was performed including only those 36 sites which reported data for the entire study period, with results similar to the overall data (see Appendix A Table A1 and Table A2).

Table 5 shows the analysis of factors potentially prognostic for meeting their accrual targets in Year 4. No factor was significant in univariable or multivariable models.

## 4. Discussion

Since the formation of 3CTN as a pan-Canadian cancer network, there has been an increase in academic cancer clinical trial recruitment. From 2014 to 2018, a total of 11,275 patients were enrolled in Portfolio trials, with 3907 patients recruited in the last fiscal year, which was an increase of 59.6% above pre-3CTN baseline yearly totals, exceeding the recruitment target for the period. This was accomplished despite challenges due to declining numbers of Portfolio Phase III and other large study population randomized clinical trials during the same period.

There were three factors that correlated with successful recruitment: time period, region and number of baseline trials. Centres with lower numbers of baseline trials had greater increases in accrual, whereas centres with larger numbers of trials had less of an increase. 3CTN support may have had less of an impact on those centres that had larger numbers of existing trial staff and infrastructure compared to smaller centres. However, the number of trials and trial personnel could not continue to expand capacity limits. Further investigation into this ceiling effect is required.

The lack of association between funding and achieving accrual targets was surprising. It is possible that sites may have secured new funding for their trial units outside of 3CTN. In addition, it is possible that the limited envelope of core and incentive funding was not sufficient to independently improve recruitment to ACCTs. The CAN 22 M total budget was substantial and unprecedented, but less than the CAN 36 M original budget developed based on adaption of the UK model.

Improved communication, best practices implementation and other 3CTN activities to improve trial performance may have also contributed to recruitment success across the Network. Improved connections and collaborative problem solving among Network centres are among advancements reported by sites and observed during Network-wide meetings. Smaller centres with more limited resources to conduct trials were linked to supports from larger centres to increase clinical trial activity. Some NCCs allocated core funding to NACCs to hire staff, while others provided educational opportunities, shared resources and coordinated activities, such as research ethics board submissions. These changes allowed smaller centres to increase their accrual by a larger percentage compared to larger centres, although total numbers were greater for larger centres with established clinical trial programs and patient catchment areas. Whether to prioritize support for smaller centres to increase their trial activity and accrual, which may allow patients more access to clinical trials, or larger centres which can accrue more patients in absolute terms, is an ongoing debate.

3CTN provided Network sites with access to clinical research professionals to share best practices, tools, and templates. In addition, 3CTN worked with stakeholders to develop and implement initiatives to address challenges in the clinical trial environment. These initiatives included supporting the roll out of clinical trials management systems, development of a recruitment best practices repository and a clinical trial education and awareness campaign. Although it will require more time to quantify the impact, these ACCT-focused improvements are expected to contribute to a more efficient and high-quality research environment for all clinical trials.

There was regional variation in attaining accrual targets. Most regions saw increased clinical trial activity above pre-3CTN baseline; however, some were unable to meet recruitment targets, while others did not sustain baseline trial activity. Twelve sites were delayed in joining the Network and may benefit from additional r time to improve trial recruitment activities. Certain regions had smaller populations and trial opportunities and capacities to participate in more trials. Core funding amounts were variable for each centre, determined by the size of the population served and availability of funds. Access to 3CTN core funding varied across provinces, partly due to available funding from the provincial funder, and partly due to the funding model based on patient population. For example, Network cancer centres located in Quebec did not receive core funding directly; instead, centralized roles were created to support sites. A more targeted approach to improving and sustaining capacity for trial conduct at centres is needed.

There are several limitations to our analysis. Unfortunately, pre-3CTN baseline recruitment data from 2011–2013 were not collected on a trial-by-trial basis, and this has limited the ability to carry out more in-depth analysis of changes in individual trials and trial portfolios over time. The reliance on trial information from trial registries and other public sources to review and supplement data provided by member cancer centres limits understanding of trial complexity. Although 3CTN membership reflects the majority of sites conducting cancer trials in Canada, the total number of sites is relatively small, and the evaluation timeframe is relatively short, resulting in somewhat limited statistical sample size and power. Furthermore, the focus was on academic multicentre trials, which may not represent the broader conduct of clinical trials, which would include single centre studies and industry-sponsored trials.

The UK experience has shown that when adequately funded, a coordinated and managed approach to clinical research is highly successful and can be sustained in the long term [13]. 3CTN is the only Canadian network that provides direct support to clinical trial units to conduct ACCTs, and met its key objectives to increase trial recruitment and performance, and achieved greater integration across clinical trial centres through its activities. Although 3CTN met its overall objectives and its initial success is encouraging, support for ACCT conduct needs to be sustained. Insufficient support for clinical trial units remains a challenge and the resulting focus on industry sponsored studies continues to be concerning [14]. 3CTN has developed the structure, platforms and linkages needed to deliver on its objectives and with time will adapt and evolve into a more sustainable network. 3CTN will continue to support ACCT and Network sites, monitor performance over time, assess for factors that contribute to successful ACCT and seek continued funding to ensure the success of the Network, resulting in better trial access and outcomes for patients.

## Figures and Tables

**Table 1:** Network site characteristics.

Parameter	Analysis	Result
Sites by Province	N (% of total)	
Alberta		2 (4.1)
British Columbia		5 (10.2)
Manitoba		1 (2.0)
New Brunswick		3 (6.1)
Newfoundland and Labrador		1 (2.0)
Nova Scotia		2 (4.1)
Ontario		24 (49.0)
Prince Edward Island		1 (2.0)
Quebec		10 (20.4)
Funding	N (%) Core Funded	27 (55.1)
Site Type	N (%) NACC *	35 (71.4)
Size	N (%) Large	12 (24.5)
	Medium	8 (16.3)
	Small	29 (59.2)
Number of Clinical Trial Staff	Median (IQR) range	5 (2.5–28.3), 0.4–280

* Network Affiliated Cancer Centres (NACC).

**Table 2:** Network site data and Portfolio trial composition analysis outcomes.

Parameter	Analysis	Pre-3CTN Baseline ^†^	Year 4
Annual Accrual	Median (IQR), range	21 (8–60), 1–396	22 (9–85), 0–511
Accrual as % of Baseline	Median (IQR), range	NA	160 (−18.2, 175), 0–1200
Number of Academic Trials per Site	Median (IQR), range	7 (3–27), 0–98	10 (5–30), 1–112
Accrual as % of Target	Median (IQR), range	NA	106 (−45.5, 175), 0–611
Number of Sites that Met Target	N (%) Yes	NA	25 (51.0)
**Portfolio Trial Composition**			
Sample size per trial	Median (IQR), range	154 (58–520), 5–5872	150 (61–448), 9–164,946
Trials with sample size 500+	N (%)	26	22
Number of Phase III trials	N (%)	38.9	29.5
Number of Patients Recruited/Trial	Median (IQR), range	NA	42 (0–20), 0–265

^†^ pre-3CTN baseline is for the period of 1 April 2013–31 March 2014.

**Table 3:** Quarterly accrual at Network sites.

Time Period	N	Median (IQR) Accrual	Mean Accrual	Sum Accrual across Sites	Median (IQR) % Change from Pre-3CTN	N (%) ≥Target	Mean (sd) Change ^†^ from Pre-3CTN	Median (IQR) % Change ^†^ from Pre-3CTN
**Y1Q3**	36	4 (0.5–17.5)	12.2	440	−29.3 (−94.9, 7.9)	4 (11.1)	−0.6 (5.1)	−0.3 (2.5, 0.5)
**Y1Q4**	36	3 (1–20)	11.9	430	−28.2 (−63.6, 20.5)	7 (19.4)	−0.9 (8.3)	−1.1 (−3.4, 1.4)
**Y2Q1**	36	4 (1–14.5)	15.6	561	7.3 (−82.3, 69.3)	12 (33.3)	2.8 (10.8)	0.5 (−1.9, 4.0)
**Y2Q2**	36	4 (0.5–14.5)	13.8	498	−16.7 (−92.6, 46.5)	9 (25.0)	1.0 (7.8)	−0.3 (−2.3, 2.9)
**Y2Q3**	47	4 (0–16)	13.1	618	−34.6 (−100, 14.3)	8 (17.0)	0.3 (8.9)	−0.3 (−2.8, 2.5)
**Y2Q4**	48	6 (0–21.5)	14.6	701	−5.6 (−100, 78.4)	15 (31.3)	1.9 (10.5)	−0.3 (−2.0, 3.6)
**Y3Q1**	49	10 (2–30)	22.1	1083	50.0 (−33.3, 180.7)	25 (51.0)	9.6 (17.8)	3.5 (−0.3, 11.0)
**Y3Q2**	49	11 (2–32)	21.9	1075	69.2 (−15.6, 200.0)	29 (59.2)	9.4 (17.8)	1.5 (−0.8, 16.3)
**Y3Q3**	49	9 (3–26)	19.9	976	33.3 (−16.3, 185.7)	21 (42.9)	7.4 (17.5)	2.5 (−0.5, 8.5)
**Y3Q4**	49	7 (1–22)	20.1	986	50.0 (−20.0, 204.8)	25 (51.0)	7.6 (21.2)	1.0 (−0.3, 10.8)
**Y4Q1**	49	8 (2–22)	23.6	1157	73.3 (−7.7, 234.8)	28 (57.1)	11.1 (22.8)	3.5 (−0.5, 11.8)
**Y4Q2**	49	6 (2–23)	20.1	986	35.5 (−45.9, 156.4)	23 (46.9)	7.6 (18.2)	1.8 (−1.8, 8.5)
**Y4Q3**	49	4 (1–15)	17.2	844	15.6 (−50.0, 100.0)	20 (40.8)	4.7 (15.4)	0.5 (−2.8, 5.0)
**Y4Q4**	49	4 (2–22)	18.8	920	14.3 (−40.0, 114.6)	19 (38.8)	6.3 (25.2)	1.0 (−1.8, 3.5)

^†^ absolute change from pre-3CTN quarterly estimate.

**Table 4:** Prognostic factors of change in accrual (repeated measures) % change. No interaction between region and time (*p* = 0.80) in multivariable model.

Factor	Value	Estimate (SE)	*p*-Value
**Region ^†^**	Western Canada	48.8 (65.8)	0.023
	Ontario	136.7 (55.2)	
	Quebec	5.6 (68.0)	
	Atlantic Canada	Reference	
**Core Funding**	Yes vs. No	20.3 (38.2)	0.6
Site Type *	NACC vs. NCC	120.9 (41.4)	0.005
**Size**	Large	−138.1 (44.4)	0.001
	Medium	−158.0 (51.7)	
	Small	Reference	
**Number of Baseline Trials**	/Trial	−3.2 (0.9)	<0.001
**Number of Trials Staff**	/Person	−0.9 (0.5)	0.052
**Multivariable Analysis**			
**Time Period**	/quarter	17.2 (4.8)	<0.001
**Region ^†^**	Western Canada	114.2 (67.9)	0.029
	Ontario	160.2 (54.8)	
	Quebec	49.5 (75.8)	
	Atlantic Canada	Reference	
**Core Funding**	Yes vs. No	43.2 (48.5)	0.38
**Number of Baseline Trials**	/Trial	−3.8 (0.9)	<0.001

^†^ Region, Western Canada includes British Columbia, Alberta and Manitoba; Atlantic Canada includes Nova Scotia, New Brunswick, Newfoundland and Labrador, and Prince Edward Island. * Site type, Network Cancer Centre (NCC); Network Affiliated Cancer Centre (NACC).

**Table 5:** Prognostic factors of meeting Year 4 (1 April 2017–31 March 2018) accrual target.

Factor	Value	Odds Ratio (95% CI)	*p*-Value
**Region ^†^**	Western Canada	1.5 (0.2, 13.2)	0.44
	Ontario	3.5 (0.6, 21.8)	
	Quebec	3.8 (0.5, 29.8)	
	Atlantic Canada	Reference	
**Core Funding**	Yes vs. No	0.6 (0.2, 1.7)	0.31
Site Type *	NACC vs. NCC	2.4 (0.7, 8.6)	0.18
**Size**	Large	0.4 (0.1, 1.7)	0.15
	Medium	0.2 (0.1, 1.2)	
	Small	Reference	
**Number of Baseline Trials**	/Trial	0.97 (0.94, 1.00)	0.073
**Number of Trials Staff**	/Person	0.97 (0.93, 1.00)	0.081
**Multivariable Model**			
**Region ^†^**	Western Canada	2.2 (0.2, 23.6)	0.44
	Ontario	4.4 (0.7, 29.3)	
	Quebec	3.7 (0.3, 42.6)	
	Atlantic Canada	Reference	
**Core Funding**	Yes vs. No	0.8 (0.2, 4.1)	0.78
**Number of Baseline Trials**	/Trial	0.97 (0.94, 1.01)	0.11

^†^ Region, Western Canada includes British Columbia, Alberta and Manitoba; Atlantic Canada includes Nova Scotia, New Brunswick, Newfoundland and Labrador, and Prince Edward Island. * Site type: Network Cancer Centre (NCC); Network Affiliated Cancer Centre (NACC).

## Data Availability

The data presented in this study are contained within this article.

## Appendix A

**Table A1:** Quarterly accrual at Network sites—subgroup of those *N* = 36 followed for entire time period.

Time period	*N*	Median (IQR)	Mean	Sum Accrual Across Sites	Median (IQR) % Change from Pre-3CTN	*N* (%) ≥Target	Mean (sd) Change ^†^ from Pre-3CTN	Median (IQR) % Change ^†^ from Pre-3CTN
		Accrual	Accrual					
Y1Q3	36	4 (0.5–17.5)	12.2	440	−29.3 (−94.9, 7.9)	4 (11.1)	−0.6 (5.1)	−0.3 (2.5, 0.5)
Y1Q4	36	3 (1–20)	11.9	430	−28.2 (−63.6, 20.5)	7 (19.4)	−0.9 (8.3)	−1.1 (−3.4, 1.4)
Y2Q1	36	4 (1–14.5)	15.6	561	7.3 (−82.3, 69.3)	12 (33.3)	2.8 (10.8)	0.5 (−1.9, 4.0)
Y2Q2	36	4 (0.5–14.5)	13.8	498	−16.7 (−92.6, 46.5)	9 (25.0)	1.0 (7.8)	−0.3 (−2.3, 2.9)
Y2Q3	36	3.5 (0.5–16.5)	14.6	524	−23.6 (−92.3, 28.0)	7 (19.4)	1.7 (8.2)	−0.3 (−2.3, 3.1)
Y2Q4	36	5.5 (0.5–21.5)	15.6	561	4.0 (−92.3, 84.5)	13 (36.1)	2.8 (8.6)	0.6 (−1.3, 4.9)
Y3Q1	36	12.5 (2–30)	24.8	892	66.1 (−19.2, 271.4)	21 (58.3)	12.0 (19.9)	6.3 (−0.3, 12.0)
Y3Q2	36	11 (2–33.5)	24.9	898	92.5 (−12.4, 300.0)	23 (63.9)	12.1 (19.8)	3.6 (−0.6, 19.3)
Y3Q3	36	9.5 (3–26)	22.2	798	35.9 (−19.2, 213.0)	15 (41.7)	9.3 (19.5)	2.5 (−0.3, 10.3)
Y3Q4	36	7.5 (1–23)	23.1	832	42.3 (−28.4, 221.5)	17 (47.2)	10.3 (23.1)	0.9 (−0.6, 11.4)
Y4Q1	36	8 (2–24.5)	24.8	893	72.9 (−20.5, 235.6)	21 (58.3)	12.0 (25.3)	3.1 (−0.4, 11.1)
Y4Q2	36	5.5 (2–19.5)	21.3	766	32.4 (−55.5, 156.7)	16 (44.4)	8.5 (20.6)	1.8 (−1.9, 8.0)
Y4Q3	36	5 (1–16.5)	19.3	693	27.3 (−52.8, 106.4)	16 (44.4)	6.4 (17.4)	0.6 (−2.3, 5.6)
Y4Q4	36	4 (2–23.5)	20.8	748	7.1 (−52.8, 110.1)	13 (36.1)	8.0 (29.1)	0.5 (−1.9, 3.6)

^†^ absolute change from pre-3CTN quarterly estimate.

**Table A2:** Prognostic factors of change in accrual (repeated measures) % change—subgroup of sites completed all time points. No interaction between region and time (*p* = 0.54) in multivariable model.

Factor		Estimate (SE)	*p*-Value
Region ^†^	Western Canada	36.6 (73.2)	0.067
	Ontario	130.8 (62.5)	
	Quebec	NA	
	Atlantic Canada	Reference	
Core Funding	Yes vs. No	−5.2 (49.4)	0.92
Site Type *	NACC vs. NCC	122.7 (50.4)	0.021
Size	Large	−148.2 (53.7)	0.006
	Medium	−168.2 (62.4)	
	Small	Reference	
Number of Baseline Trials	/Trial	−3.4 (1.0)	0.001
Number of Trials Staff	/Person	−0.9 (0.5)	0.1
Multivariable Analysis			
Time Period	/quarter	20.1 (5.8)	<0.001
Region ^†^	Western Canada	23.8 (112.8)	0.13
	Ontario	136.3 (88.3)	
	Quebec	NA	
	Atlantic Canada	Reference	
Core Funding	Yes vs. No	10.6 (67.5)	0.88
Site Type	NACC vs. NCC	−338.9 (183.1)	0.076
Size	Large	78.6 (204.8)	0.93
	Medium	21.6 (87.8)	
	Small	Reference	
Number of Baseline Trials	/Trial	−14.3 (4.0)	0.001
Number of Trials Staff	/Person	2.4 (1.0)	0.018

^†^ Region, Western Canada includes British Columbia, Alberta and Manitoba; Atlantic Canada includes Nova Scotia, New Brunswick, Newfoundland and Labrador, and Prince Edward Island. * Site type, Network Cancer Centre (NCC); Network Affiliated Cancer Centre (NACC).
